# Refinery wastewater treatment via a multistage enhanced biochemical process

**DOI:** 10.1038/s41598-021-89665-8

**Published:** 2021-05-13

**Authors:** Chunhua Wang, Zijian Chen, Yuanhua Li, Kejun Feng, Zhongli Peng, Yongjuan Zhu, Xiaofang Yang

**Affiliations:** 1 School of Chemistry and Materials Engineering, Huizhou University, 3# Building, Huizhou, 516007 People’s Republic of China; 2 CNOOC Huizhou Petrochemical Company, Huizhou, 516018 People’s Republic of China

**Keywords:** Biological techniques, Environmental sciences

## Abstract

Petroleum refinery wastewater (PRWW) that contains recalcitrant components as the major portion of constituents is difficult to treat by conventional biological processes. An effective and economical biological treatment process was established to treat industrial PRWW with an influent COD of over 2500 mg L^−1^ in this research. This process is mainly composed of internal circulation biological aerated filter (ICBAF), hydrolysis acidfication (HA), two anaerobic–aerobic (A/O) units, a membrane biological reactor (MBR), and ozone-activated carbon (O_3_-AC) units. The results showed that, overall, this system removed over 94% of the COD, BOD_5,_ ammonia nitrogen (NH_4_^+^-N) and phosphorus in the influent, with the ICBAF unit accounting for 54.6% of COD removal and 83.6% of BOD_5_ removal, and the two A/O units accounting for 33.3% of COD removal and 9.4% of BOD_5_ removal. The degradation processes of eight organic pollutants and their removal via treatment were also analyzed. Furthermore, 26 bacteria were identified in this system, with *Proteobacteria* and *Acidobacteria* being the most dominant. Ultimately, the treatment process exhibited good performance in degrading complex organic pollutants in the PRWW.

## Introduction

Petroleum refineries produce products such as gasoline, diesel, and lubricating oils that are integral to a nation's economic development. However, petroleum refinery wastewater (PRWW) contains many pollutants, including heavy metals, volatile phenols, and polycyclic aromatic hydrocarbons^1^, which are difficult to eliminate during regular wastewater treatment processes^2^. As such, researchers have developed a number of alternative wastewater treatment techniques aimed at removing these compounds, including biological techniques^3^, adsorption-based techniques^4^, chemical-oxidation techniques^5^, and flotation- and oxidation-based techniques^6^. Biological techniques—for example, traditional anaerobic/aerobic (A/O) processes, membrane bioreactor (MBR) systems, or biological aerated filter (BAF) systems—are environmentally friendly, but they often lack the capacity to remove all pollutants in PRWW with an influent COD above 2000 mg L^−1^^7^. Given this limitation, a number of combined treatment processes have been proposed in recent years^8–11^. Yang et al.^8^ combined microaerobic hydrolysis–acidification (MHA) and anoxic–oxic (A/O) units to treat petrochemical wastewater, achieving a COD removal efficiency of 75% and an ammonium removal rate of 94% at a hydraulic retention time (HRT) of 20 h. Liu et al.^9^ combined an up-flow anaerobic sludge blanket (UASB) and immobilized biological aerated filters (IBAFs) to treat heavy oil wastewater containing dissolved recalcitrant organic compounds and low level of nitrogen and phosphorus. Their approach enabled the removal of 74% of COD, 94% of NH_4_^+^-N, and 98% of suspended solids from the discharged wastewater. Finally, Wu et al.^10^ developed a sequence process that combined hydrolysis acidification (HA) and anoxic–oxic (A/O) units to treat petrochemical wastewater with a COD of 480 mg L^−1^. Their results showed that their developed approach was able to reduce the COD value to 60 mg L^−1^, in addition to reducing the presence of five other organic compounds (indene, 1,3-dioxolane, 2-pentanone, 2-chloromethyl-1,3-dioxolane and ethylbenzene). Despite their successes, each of these studies were constrained by certain limitations. For instance, the above techniques were tested at a lab scale using influent with a COD of < 1500 mg L^−1^, which is much lower than the COD typically found in industrial influent (> 2000 mg L^−1^)^12^. Furthermore, despite combining 2 or 3 biological treatment units, the above approaches were unable to sufficiently treat refractory organic macromolecular pollutants in the influent. For example, Wu et al.^10^ still detected low acute toxicity in the effluents in their luminescent bacteria assay. Thus, there is still a pressing need for an effective and economical biological process that is capable of bringing heavily contaminated PRWW into compliance with discharge standards.

According to the refractory characteristics of PRWW, we examine a petrochemical wastewater treatment system consisting of an ICBAF tank, an HA tank, two sets of A/O tanks, an MBR, and O_3_-AC units. Based on the traditional A/O process, the ICBAF tank and the HA tank were pre treated to improve the biodegradability of macro molecules and reduce the load of the A-O unit. The MBR unit and the O_3_-AC unit were installed to enhance the degradation of macro-molecular refractory organics, so that the sewage can meet the national discharge standard. Specifically, this research was aimed at achieving three key objectives: (1) to evaluate the system’s performance with respect to the removal of organics, ammonia, nitrogen, and phosphorus; (2) to analyze the degradation process of the main organics in each tank; and (3) to identify the bacterial community of the sludge in each biological tank.

## Results and discussion

### Performance of the wastewater treatment process

#### The biological degradation performance in the PRWW

**Figure 1:**
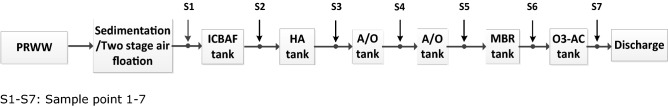
Sampling points of the PRWW process. S1–S7: Sample point 1–7.

Table 1 shows the macro organic pollutant concentrations in wastewater samples collected at seven points throughout the PRWW treatment process (Fig. 1). The biological degradation performance is closely related to the COD, the BOD_5_, the BOD_5_/COD, and the C: N: P values.

**Table 1:** Analysis results of six indexes at each sampling point (mg L^−1^).

Items	COD	BOD_5_	BOD_5/_COD	TOC	C: N: P	VFA	Sulfide
S1	2554 ± 538	1198 ± 302	0.469	611 ± 41	257:28:1	11.67 ± 3.5	8.24 ± 5.9
S2	1159 ± 253	196 ± 58	0.169	320 ± 38	68:42:1	0.21 ± 1.5	1.87 ± 1.5
S3	1040 ± 116	114 ± 11	0.102	312 ± 29	38:35:1	0.13 ± 0.6	5.23 ± 3.3
S4	230 ± 28	7 ± 5	0.03	84 ± 24	4:25:1	ND	0.16 ± 0.1
S5	190 ± 39	2 ± 0	0.01	50 ± 3	1.5:39:1	ND	ND
S6	110 ± 19	ND	ND	47 ± 12	ND	ND	ND
S7	50 ± 2	ND	ND	20 ± 1	ND	ND	ND

*S1-S7* sample point 1–7; *ND* no detection; *COD* chemical oxygen demand; *BOD*_*5*_ Biochemical oxygen demand; *TOC* total organic carbons; *VFA* volatile fatty acids.

As shown in Table 1, the COD, BOD_5_, and the BOD_5_/COD values varied throughout the process, indicating that the wastewater had undergone different levels of biological degradation at the various sample collection points.

At the ICBAF influent (S1), the BOD_5_/COD value was 0.469, and the C:N:P ratio was 257:28:1. This nutrient ratio was conducive to the metabolism of pollutants by the microorganisms in the ICBAF unit, which resulted in a 54.6% in COD and an 83.6% decrease in BOD_5_ for hydraulic detention time (HRT) of 14 h in the ICBAF tank. This high removal efficiency indicated that the ICBAF tank played an important role in pretreating the PRWW. At the ICBAF effluent (S2), the BOD_5_/COD value was 0.169 and the C:N:P ratio was 68:42:1, indicating a significant decrease in the wastewater's biodegradability compared to the influent (S1). To improve biodegradability, an HA tank was added. Unfortunately, the BOD_5_/COD of the resultant HA effluent (S3) further declined to 0.102, which means that its biodegradability had also declined. This result is likely attributable to the large amount of oxygen in the effluent entering the HA tank from the ICBAF, as such oxygen levels would affect the anoxic environment in the HA tank. Thus, the efficiency of hydrolytic acidification is not fully realized under these conditions. The C:N:P ratio at the HA effluent (S3) was a reasonable 38:35:1. However, the carbon source quality was poor, which was not conducive to denitrification in the subsequent A/O units. At the first A/O tank effluent (S4), the COD value decreased to about 30% of its value at the first A/O influent (S3) and the BOD_5_/COD value declined to 0.03 at HRT of 24 h, indicating the COD at the first A/O tank effluent (S4) was mainly composed of refractory organics that are difficult to remove via the biochemical degradation process. In addition, the C:N:P ratio at the second A/O influent (S4) was 4: 25: 1, which indicates that the quantity and quality of the carbon source was insufficient for the subsequent biochemical treatment. At the second A/O unit (S5)—which was taken after glucose was added as an extra carbon source to improve biodegradability—the BOD_5_/COD value was 0.01 and the C:N:P ratio was 1.5:39:1 at HRT of 28.8 h. Notably, the BOD_5_/COD value could not be detected at the MBR effluent (S6), which indicates that the organics in the wastewater was almost entirely comprised of refractory organic pollutants. Similarly, the C:N:P ratio could also not be detected at the MBR effluent (S6). This result indicates lower levels of carbon and higher levels of nitrogen in S6, and thus, that the effluent is not suitable for biological treatment. To remove the refractory organics, the effluent was then sent to an O_3_-AC tank, where the COD and TOC concentrations further decreased significantly. Finally, neither the BOD_5_/COD value nor the C:N:P ratio were detected at the O_3_-AC effluent (S7); significantly, however, a COD value of 50 ± 2 mg L^−1^ and a TOC value of 20 ± 1 mg L^−1^ were obtained, which both satisfy the pollutant discharge standards for China's petrochemical industry.

#### The concentration of the VFA and sulfide in the PRWW

As shown in Table 1, the volatile fatty acid concentration (VFA) at the ICBAF influent (S1) was 11.67 ± 3.5 mg L^−1^ and 0.21 ± 1.5 mg L^−1^ at the ICBAF effluent (S2), which indicates that most of the VFAs were removed under aerobic conditions in the ICBAF tank. The VFA concentration further fell to 0.13 ± 0.6 mg L^−1^ after treatment in the HA unit (S3), and was completely eliminated following treatment in the first A/O unit (S4). This result shows that the combined process examined in this research is effective for treating the industrial petroleum wastewater.

Table 1 also shows changes in the sulfide concentration between S1 and S7. The sulfide concentration at the ICBAF influent (S1) was 8.24 ± 5.9 mg L^−1^, but it dropped to 1.87 ± 1.5 mg L^−1^ after treatment in the ICBAF tank (S2). This dramatic decrease likely occurred due to the biodegradation and aeration stripping processes that occur in the ICBAF tank^13^. The sulfide concentration then increased to 5.23 ± 3.3 mg L^−1^ in the HA tank (S3), due to the sulfate and oxides of sulfur being reduced to sulfide as a result of this tank's anaerobic nature at HRT of 25 h. However, the sulfide was completely oxidized to sulfate after the subsequent two A/O units, which was evidenced by the absence of sulfide in the samples taken after the second A/O unit. Thus, this process successfully produced effluent that was safe to discharge.

### Characterization of pollutants in the treatment process

#### Degradation of ammonia nitrogen

Biological nitrogen removal process consists of two steps: (1) oxidizing the NH_4_^+^-N into nitrate-nitrogen, nitrite-nitrogen, or some other nitrogen form, which is a critical step in the denitrification in PRWW^14^; and (2) denitrifying the nitrate or nitrite into nitrogen gas.

**Figure 2:**
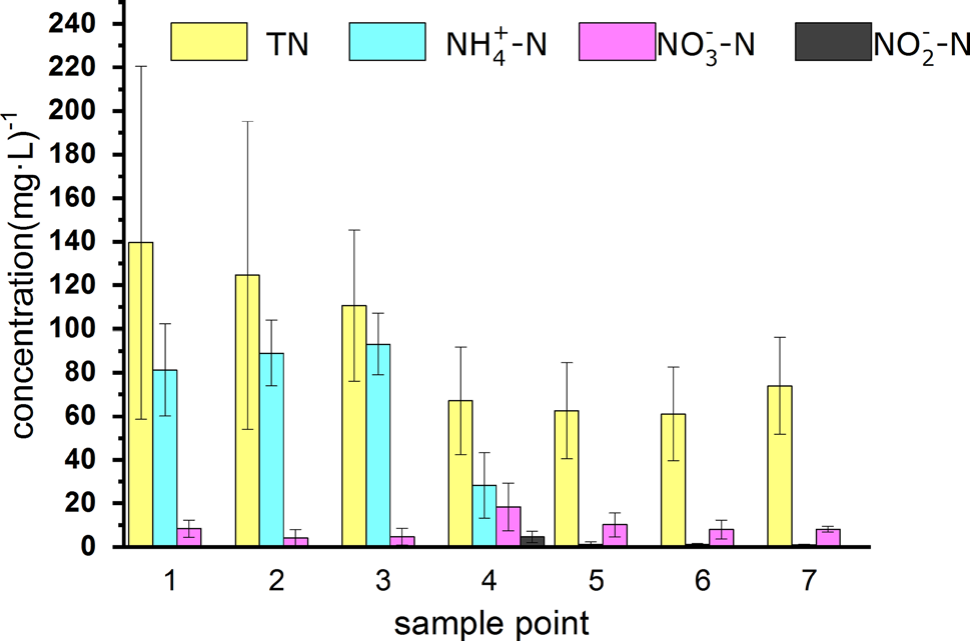
Variation of TN, NH_4_^+^-N, NO_3_-N and NO_2_-N at different sample point.

As shown in Table 2, NH_4_^+^-N removal was similar in the ICBAF and HA tanks, with results of 81.2 ± 21 mg L^−1^(ICBAF influent, S1), 89 ± 15 mg L^−1^ (ICBAF effluent, S2), and 93 ± 14 mg L^−1^ (HA effluent, S3) being obtained, respectively. These results were due to the ammoniation of microorganisms in the PRWW, which caused some degree of fluctuation in the TN and the NH_4_^+^-N, as shown in Fig. 2. While the concentrations of TN and NH_4_^+^-N decreased significantly after the first A/O process (S4), the concentrations of nitrate (NO_3_^−^) and nitrite (NO_2_^−^) increased significantly. This variation in parameters between the first A/O influent (S3) and effluent (S4) indicated that significant ammoniation and nitrification had occurred in the process at HRT of 24 h and sludge retention time (SRT) of 45 d. After the second A/O process (S5), the TN concentration decreased to 62.7 ± 22 mg L^−1^, and the concentrations of NH_4_^+^-N, NO_3_^–^N and NO_2_^–^N also declined significantly compared with the corresponding indexes in the first A/O effluent (S4). These results revealed that there was significant nitrification activity and poor ammonification and denitrification at HRT of 28.8 h and SRT of 45 d in the second A/O unit. Unfortunately, the nitrification and denitrification had no effect in the subsequent MBR and O_3_-AC tanks. As such, the concentrations of TN and NO_3_^–^N in the effluent in the O_3_-AC tank were 22.4 ± 3 mg L^−1^ and 6.1 ± 1.3 mg L^−1^, respectively.

**Table 2:** Analysis results of macro organic pollutants at each sampling point (mg L^−1^).

Items	TN	NH_4_^+^-N	NO_3_^–^N	NO_2_^–^N	TP
S1	139.6 ± 81	81.2 ± 21	8.4 ± 4	ND	4.7 ± 3.2
S2	124.6 ± 70.6	89 ± 15	4.2 ± 3.7	ND	2.9 ± 2.5
S3	110.7 ± 34.5	93 ± 14	4.7 ± 3.7	ND	3 ± 2.8
S4	67.1 ± 24.7	28.3 ± 15	18.3 ± 11	4.6 ± 2.6	1.7 ± 1.7
S5	62.7 ± 22	1.1 ± 0.4	10.1 ± 3	0.03 ± 0.03	1.3 ± 1.5
S6	24.1 ± 2.4	0.5 ± 0.1	4 ± 1.4	ND	0.3 ± 0.04
S7	22.4 ± 3	ND	6.1 ± 1.3	ND	0.2 ± 0.1

*S1-S7* Sample point 1–7; *ND* no detection; *TN* total nitrogen; *NH*_*4*_^+^*-N* ammonia nitrogen; *NO*_*3*_*-N* nitrate nitrogen; *NO*_*2*_*-N* nitrite nitrogen; *TP* total phosphorus.

#### The removal of phosphorus

As shown in Table 2, the total phosphorus (TP) concentration decreased from 4.7 ± 3.2 mg L^−1^ to 0.2 ± 0.1 mg L^−1^ throughout the wastewater treatment process (i.e. between S1 and S7), which confirms that the phosphorus was utilized effectively by the microorganism in the biological treatment units. Biological dephosphorization consists of two steps: (1) Under anaerobic conditions, polyphosphate is hydrolyzed by englobing electron donors and absorbing the readily biodegradable COD to synthesize poly-β-hydroxy butyrate (PHB), which serves as the cell's energy storage material. (2) Under aerobic conditions, where the free oxygen functions as an electron acceptor, the phosphorus bacteria oxidizes the PHB to produce energy. The phosphorus bacteria uses the energy generated in these anaerobic and aerobic conditions to assimilate the phosphate from the wastewater in order to synthesize high-energy ATP and polyphosphate, which is then stored in cells^15^. The DO concentration was 3–4 mg L^−1^ in the ICBAF tank, aerobic-anoxic-anaerobic areas were formed in the biofilm from the surface to the inside of the filter. Since the aerobic phosphorus uptake is greater than the anaerobic phosphorus release, the TP concentration in the effluent at S2 was much lower than at S1. Moreover, Chemical precipitation also played an important role in the removal of phosphorus^17^. In the biofloccula and biofilm of each biochemical reaction unit, the pH value increased with denitrification, which promoted the formation of calcium hydroxyphosphate and struvite precipitation. These precipitates accumulated in sludge or biofilm. With back-washing (in ICBAF and MBR) and sludge discharge (in two A/O units), the phosphorus containing sediment was discharged from the wastewater treatment process. Under the synergistic effect of biological phosphorus removal and chemical precipitation, the TP concentration decreased once again as it passed through the two A/O units (S4, S5), the MBR tank (S6) and the O_3_-AC process (S7).

### Characterization of organic pollutants in the process

Eight organic pollutants—namely, organic acids, esters, alcohols, heterocyclic compounds, alkanes, aromatic hydrocarbons, aldehydes and ketones, and phenols—were analyzed with respect to four attributes: (1)quantity, (2)carbon chain length range, (3)relative molecular weight distribution range, and (4)concentration. The samples were collected from the sedimentation/two stage air floation effluent (S1) to the O_3_-AC effluent (S7), and the results are shown in Table 3.

**Table 3:** Organic pollutants in wastewater samples collected from different treatment units in a petroleum refinery wastewater plant.

Sample point	Pollutant type	Organic acids	Esters	Alcohols	Heterocyclic compound	Alkanes	Aromatic hydrocarbons	Aldehydes and ketones	Phenols	Total
S1	QP	28	11	11	13	5	9	9	3	89
	CCL	C_2_-C_20_	C_10_-C_20_	C_5_-C_15_	C_5_-C_11_	C_8_-C_14_	C_8_-C_11_	C_6_-C_15_	C_6_-C_10_	
	MW	60–312	156–310	86–224	86–191	108–268	106–142	82–220	94–154	
	C	648	158	130	110	55	12	65	97	
S2	QP	12	20	8	9	3	5	1	1	59
	CCL	C_6_-C_20_	C_9_-C_20_	C_9_-C_17_	C_6_-C_16_	C_10_-C_12_	C_14_-C_19_	C_12_	C_6_	
	MW	142–294	156–334	140–244	113–244	136–168	186–256	180	94	
	C	90	228	90	98	38	18	7	0.7	
S3	QP	13	18	8	9	7	2	1		58
	CCL	C_6_-C_20_	C_9_-C_20_	C_9_-C_17_	C_6_-C_16_	C_10_-C_15_	C_14_-C_19_	C_12_		
	MW	142–294	156–334	140–244	113–244	136–168	186–256	180		
	C	84.3	177	82.6	97	51	11	6.7		
S4	QP	5	10	7	20	8	1	7		58
	CCL	C_14_-C_20_	C_15_-C_26_	C_10_-C_20_	C_4_-C_22_	C_10_-C_27_	C_16_	C_9_-C _21_		
	MW	244–296	240–372	152–312	102–385	138–378	210	152–358		
	C	15.5	12.7	15	46.6	15	0.8	14		
S5	QP	5	12	7	15	13	1	4		57
	CCL	C_5_-C_18_	C_16_-C_26_	C_5_-C_20_	C_4_-C_22_	C_12_-C_21_	C_16_	C_10_-C _16_		
	MW	116–284	252–394	86–310	102–346	170–296	210	156–314		
	C	17	15.6	4	37.4	11.2	1	2		
S6	QP	7	13	5	10	17	1	4		57
	CCL	C_8_-C_20_	C_16_-C_30_	C_5_-C_27_	C_4_-C_22_	C_11_-C_26_	C_25_	C_10_-C _21_		
	MW	178–312	266–438	86–416	102–346	156–364	344	156–318		
	C	15	10.6	2.6	13.5	10.8	0.3	1.3		
S7	QP	3	7	8	6	27	5	2		58
	CCL	C_16_-C_22_	C_16_-C_26_	C_5_-C_20_	C_4_-C_18_	C_4_-C_28_	C_11_-C_16_	C_5_-C_7_		
	MW	256–338	268–394	86–314	102–281	90–382	142–206	84–148		
	C	3.3	5	4.5	3.3	15.2	2.8	0.8		

*S1-7* sample point 1–7; *QP* quantity of pollutants; *CCL* carbon chain length range; *MW* relative molecular weight distribution range; concentration (mg L^−1^).

#### The degradation of organic acids

Table 3 shows that the concentration and the quantity of organic acids decreased, while the molecular structure of organic acids became complex throughout the process. The most significant decrease in the concentration of organic acids occurred in the ICBAF tank and in the first A/O unit, which was due to the aerobic degradation of the volatile fatty acids (VFA) and small molecular organic acids (such as butyric acid, pentanoic acid, caproic acid, 3-methylvaleric acid, and heptanic acid). According to our prior work^16^, the aerobic degradation pathways include α-oxidation, β-oxidation, combined oxidation of α and β, and aromatisation. Meanwhile, the concentration and the quantity of the organic acids basically remained unchanged after passing through the first A/O unit. Furthermore, there were no clear rules governing the changes to the molecular structures throughout the process, which indicates that the microorganisms had failed to degrade the complex organic acids. These complex organic acids were eliminated in the O_3_-AC tank, where the concentration of organic acids was 3.29 mg L^−1^. This result confirms that the treatment process was able to nearly fully degrade the organic acids in the PRWW.

#### The degradation process of alcohols

As shown in Table 3, the indexes of alcohols varied throughout the process, with most being removed in the ICBAF tank and the first A/O unit. These two units offer oxygen-rich environments^17^ and long HRTs, which allows dehydrogenation to occur. The dehydrogenation of alcohols produces aldehydes, which lose two electrons and two hydrogen ions. As a result, the concentration of alcohols is decreased to levels that comply with the discharge standard.

#### The degradation process of esters

Table 3 shows the degradation of esters in different units. As can be seen, the concentration and quantity of esters gradually decreased throughout the process (S3 to S7). The exception to this trend occurred in the ICBAF tank (S2), where small molecules of acids (such as butyric acid, pentanoic acid, caproic acid, 3-methylvaleric acid, and heptanic acid, etc.) and alcohols (such as 3-penten-2-ol, 2-butoxyethanlo, etc.) formed and hydrolysed complex macromolecular esters (such as 2-methyl-2-amyl acrylate, ethyl 4-octenoate, etc.), causing an increase in both the concentration and the quantity of esters.

Nonetheless, the biochemical treatments applied in the subsequent units introduced enzymes that degraded or decomposed the esters into small molecular compounds via oxidation, reduction, and hydrolysis, among other processes. Among these treatments, enzymes produced by microorganisms played a particularly important role in severing the carboxylic acid ester bond, thereby generating small molecular carboxylic acid and alcohol^18^. Furthermore, metabolic processes, such as oxidation and conjugation, were used to transform the small molecular compounds into less toxic or non-toxic compounds.

#### The degradation process of heterocyclic compounds

Table 3 shows that the concentration of heterocyclic compounds gradually decreased throughout the treatment. However, the quantity of these compounds decreased after treatment in the ICBAF tank, but increased after treatment in the first A/O unit. The increase in the quantity and decrease in the concentration of these compounds in the first A/O unit occurred as a result of the polycyclic heterocyclic compounds (such as 2-hydroxy-2,3-cyclododecane-nitroketone, methyl 2-(2,4-dinitrophenyl) sulfonyl benzoate, etc.) being cut off into smaller molecules, which were then biodegraded.

The mechanism that governs the biological degradation of heterocyclic compounds is an electrophilic reaction in which biodegradability increases with higher π bond charge densities. There are three critical steps in the anaerobic degradation of heterocyclic compounds: (1) the partial scission of polycyclic and heterocyclic rings; (2) the cleavage of long chains; and (3) the degradation of these organics through anaerobic fermentation, which is enabled by specific enzymes contributed by microorganisms^19^. These fission products are then funneled into the tricarboxylic acid cycle through a variety of pathways, thus allowing the effluent in S7 to meet the discharge standard.

#### The degradation process of aldehydes and ketones

Table 3 shows that the aldehydes and ketones decomposed throughout the process, with concentrations decreasing from 65 mg L^−1^ at the ICBAF influent (S1) to 0.8 mg L^−1^ at the O_3_-AC effluent (S7). Notably, the concentration and quantity of aldehydes and ketones increased after treatment in the first A/O unit (S4), which was caused by the left over intermediate products of alcohol dehydrogenation. After leaving the first A/O unit, the aldehydes were further oxidised to ketoacids^17^ via α-oxidation or β-oxidation.

#### The degradation process of aromatic hydrocarbons

The concentrations of aromatic hydrocarbons at every sample point are shown in Table 3. First, the influent (in S1) was fed into the ICBAF tank; here, the aromatic hydrocarbons decreased in quantity, slightly increased in concentration, and significantly increased in molecular weight. These variations were due to the fact that it was difficult to decycle the oxidized hydrocarbons and multiple aromatic rings (such as 2-butyl-3-hexyl-1 h-indene, 1,2-diisobutylene benzene, etc.) in the effluent^20^. In addition, naphthalene, indene, and other polycyclic aromatic hydrocarbons were formed in the ICBAF by small aromatic compounds. After treatment in the HA tank (S3), the quantity and concentration of aromatic hydrocarbons in the PRWW decreased significantly. This decrease was attributable to the HA tank's anoxic ecosystem at HRT of 25 h, which enabled the enzymes to convert the aromatic substrates into an ortho- or para-cyclohexane carboxylic acid derivatives, which was followed by the cleavage of the ring^21^. After the final treatment step in the O_3_-AC tank, the concentration of aromatic hydrocarbons increased from 0.3 mg L^−1^ (the O_3_-AC influent, S6) to 2.8 mg L^−1^ (the O_3_-AC effluent, S7). This increase was due to the aromatic hydrocarbons that remained in the effluent after the partial oxidization and decomposition of the benzene ring heterocyclic compounds. Thus, the quantity and the concentration of aromatic hydrocarbons increased in the effluent exiting the O_3_-AC tank.

#### The degradation process of alkanes

The results for alkane removal are also listed in Table 3. As can be seen, the quantity of alkanes initially decreased in the ICBAF tank before increasing in the subsequent treatment steps. This result was due to portions of the alkanes (such as 1-methylcyclohexane, 1-methyl-4-(1-methyl-ethyl) cyclohexane, etc.) being degraded in the ICBAF tank, and macro-molecular long-chain alkanes and cycloalkanes (such as 1,2,3,4,4a,5,6,8a-octahydronaphthalene, etc.) remaining in the wastewater. The concentration and quantity of alkanes increased in the HA tank, which was due to the insoluble macro-molecular alkanes being hydrolyzed into soluble alkanes by extracellular enzymes within the tank's anoxic environment at HRT of 25 h. In the biochemical units (i.e. the two A/O units), the quantity of alkanes increased from 7 (at the HA effluent, S3) to 13 (at the second A/O effluent, S5), while the concentration decreased from 51 mg L^−1^ (at S3) to 11.2 mg L^−1^ (at S5). This result was due to the failure of the microorganisms to decompose the macro-molecular alkanes (i.e. 5-propyl-tridecane, 2,6,10,15-tetramethylheptadecane) when their molecular structures were quite stable. In addition, other pollutants, such as heterocyclic compounds and aromatic hydrocarbons, might decompose into small molecules made up of straight-chain alkanes and cycloalkanes at HRT of 24 h and 28.8 h in the two A/O units respectively. These small molecules were oxidized into organic acids in the addition-dehydrogenation-hydroxylation process before being decomposed into CO_2_ and H_2_O^17^ in the two A/O units and the MBR tank (S6), where the alkane concentration declined to 10.8 mg L^−1^. Since the macro-molecular alkanes were oxidized into small molecular alkanes in the O_3_-AC tank, the concentration and quantity of alkanes increased, but the relative molecular weight decreased.

In summary, the pollutant concentrations were reduced by 99.5%, with the best treatments results observed in the biochemical units. Indeed, the removal efficiency of organic acids, alcohols, aldehydes and ketones, aromatic hydrocarbons, and phenols in the biochemical units exceeded 97%. The ICBAF tank displayed good ability to decompose small-molecule pollutants, the HA tank proved highly capable of decomposing most macro-molecular compounds into degradable small-molecule substances, and the two A/O units were efficient in transforming the micro-molecular organics into biodegraded compounds. Although non-biodegradable macro-molecular materials still remained in the effluent of the MBR tank, subsequent treatment in the O_3_-AC unit's ozone environment was able to decompose most of these remaining organics^22^. Therefore, the combined wastewater treatment process examined in this study is fully capable of treating industrial petroleum wastewater such that it complies with discharge standards.

### The DHA value of the biochemical unit

Dehydrogenase enzyme activity (DHA) has been widely used to evaluate biological activity in soils, sediments, aerobic-activated sludge, and anaerobic sludge^23^ related to many of the different intracellular and specific dehydrogenase enzymes^24^ in the dehydrogenation process.

**Figure 3:**
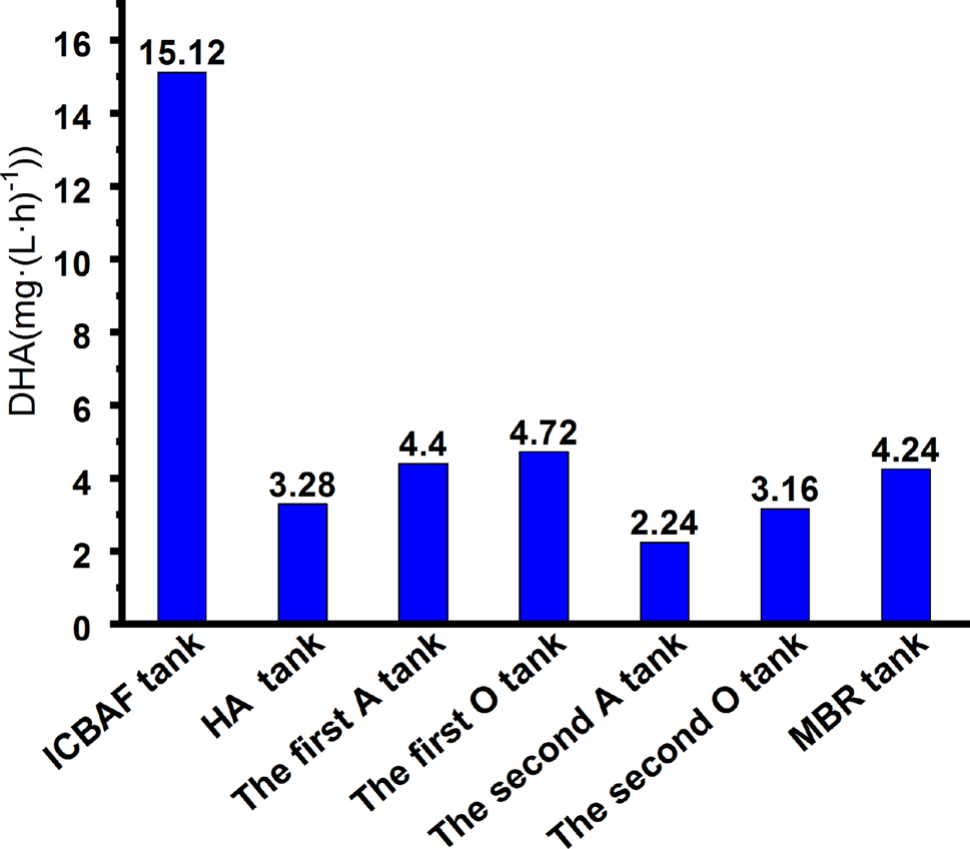
DHA of the biochemical units.

The DHA variations in the biochemical units are shown in Fig. 3. As can be seen, the highest DHA value was recorded for the ICBAF tank, at 15.12 mg (L h)^−1^, which indicates that the microorganisms within the tank were active enough to significantly reduce the COD and BOD_5_. The DHA value was 3.28 mg (L h)^−1^ in the HA tank, which indicates that the content of effective microorganisms was not large enough to hydrolyze organic compounds. The DHA values of the first A tank, the first O tank and the MBR tank were 4.4 mg (L h)^−1^, 4.72 mg (L h)^−1^, and 4.24 mg (L h)^−1^, respectively, which indicates good biodegradation of organic compounds. In contrast, the DHA values of the second A tank and the second O tank were 2.24 mg (L h)^−1^and 3.16 mg (L h)^−1^, which indicates poor efficiency with respect to pollutant degradation. Thus, the removal mechanisms of the pollutants were clarified by verifying the DHA values in the different treatment units.

### Bacterial community in the biochemical units

**Figure 4:**
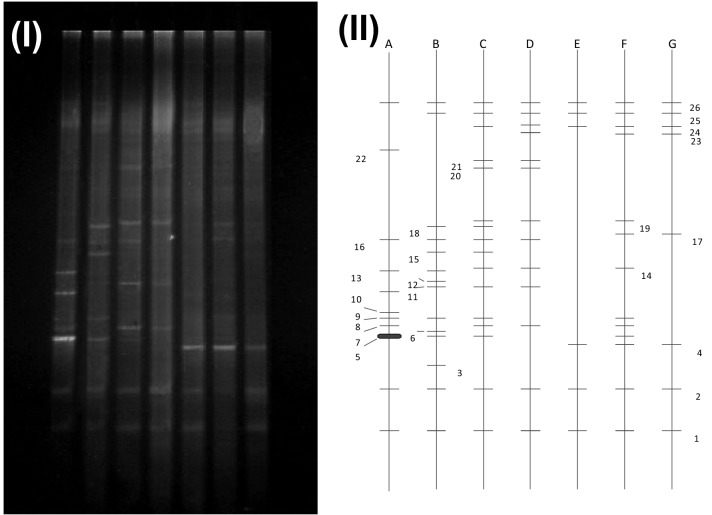
Comparison of microbial community of total bacteria in different samples (**A:** ICBAF; **B**: HA tank; **C**: the first A tank; **D**: the first O tank; **E**: the second A tank; **F**: the second O tank; **G**: MBR) Nos. 1–26 represent the light bands; the lines at the same height in II correspond to the band in I.

The PCR-DGGE technology based on 16S ribosomal DNA (rDNA) gene is a common molecular biology experiment to assess the microbial bacterial community. This technology was applied to investigate the bacterial community in different bioreactors. Because DGGE can only analyze DNA fragments with less than 500 bp, less information about phylogeny can be obtained. The 26 DGGE light bands in Fig. 4-I were excised, reamplified, sequenced, and reflected the dominant bacteria communities in the bioreactors, while Fig. 4-II denotes the different tanks in the process being using A to G. The horizontal lines in Fig. 4-II indicate the presence of a bacteria; thus, a higher density of lines indicates a higher level of bacteria in a treatment unit. Among the bioreactors, more bacteria were found in the ICBAF tank and the first A/O unit than in the MBR tank, indicating that the bacterial communities in the early biological reaction treatment units were probably the result of their different bacterial growth environments and nutrient conditions.

**Figure 5:**
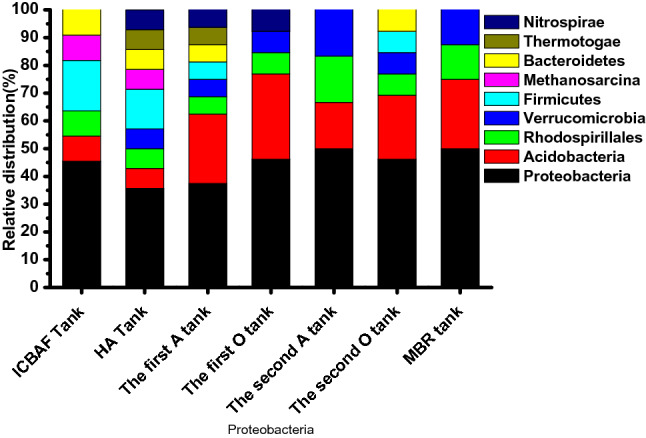
Relative abundance of the major bacterial community at phylum levels (only higher than 1% was present).

Table 4 shows the phylum of the bacteria represented by the DGGE bands, including *Proteobacteria, Acidobacteria, Firmicutes,* and *Methanosarcina*. Figure 5 shows the relative abundance of the major bacterial community at phylum levels. As can be seen, *Proteobacteria* were dominant in all bioreactors, comprising a wide variety of aerobic, anaerobic, and facultative bacteria. The *Proteobacteria* associated with active methylotrophs by virtue of their physical and/or nutritional effects^10^ were able to degrade most organic contaminants and remove biological nitrogen and phosphorous^8^. These results correspond to Miura et al. research^25^, wherein *Proteobacteria* was considered the dominant phylum in the MBR unit and played an important role in the removal of organic matter. *Acidobacteria* were also widely distributed in all bioreactors, as shown in Fig. 5 Since the *Acidobacteria* are acidophilic^26^, they are suitable for treating the high-acid, low-sulfur crude oil wastewater in this study. *Firmicutes* can produce extracellular enzymes such as cellulose, lipase, and protease, which are important for hydrolyzing and utilizing the refractory chemicals in petrochemical wastewater^8^. As shown in Fig. 5, most of the *Firmicutes* were found in the ICBAF tank, the HA tank, the first anaerobic tank, and the second aerobic tank, which was consistent with the decomposition of organic pollutants within these units. *Bacteroidetes* were found in the ICBAF tank, the HA tank, the first anaerobic tank, and the second aerobic tank. *Bacteroidetes* can break down macro-molecules such as protein, starch, cellulose, and fiber^8^ in the fermentation system, in addition to being able to degrade complex organic matter^27^. *Methanosarcina*, a hydrogenotrophic methanogen, was found in the ICBAF tank and the HA tank, which were where anaerobic respiration processes took place. The *Methanosarcina* were responsible for the production of H_2_–CO_2_, methanol, mono-, di-, and trimethylamines, acetate, and CO^28^. *Nitrospirae* were mainly found in the HA tank, the first anaerobic tank, and the first aerobic tank (Fig. 5.). *Nitrospirae* were effective in removing ammonia nitrogen^29^.

**Table 4:** Phylogenetic sequence affiliation of amplified 16S rDNA sequence excised from DGGE of the biochemical units.

Band	Organism	Phylum	Accession number	Blast similarity (%)
1	*Gluconacetobacter *sp.* T61213-21-1a*	*Rhodospirillales*	B778532.1	100
2	*Thauera *sp.* BC0187*	*Proteobacteria*	*KC166840.1*	95
3	*Soehngenia *sp.* B4119*	*Methanosarcina*	HQ133183.1	100
4	*Geobacter *sp.* KS-54*	*Proteobacteria*	EU809806.1	91
5	*Clostridiales bacterium De1161*	*Firmicutes*	HQ183782.1	100
6	*Rhodocyclaceae bacterium MBfR_NS-150*	*Proteobacteria*	JN125706.1	98
7	*Uncultured bacterium OX G09*	*Proteobacteria*	FN429550.1	100
8	*Bacteroidales bacterium M6*	*Bacteroidetes*	KC769129.1	99
9	*Firmicutes* bacterium D004025G03	*Firmicutes*	GU179831.1	100
10	*Comamonadaceae* bacterium B1-08	*Proteobacteria*	JF754519.1	99
11	*Clostridium* sp.PACOL4_36	*Firmicutes*	GQ257695.1	94
12	*Uncultured* bacterium ZBAF2-55	*Nitrospirae*	HQ682030.1	100
13	*Soehngenia* sp. L35B_140	*Methanosarcina*	JF946902.1	100
14	*Acidobacteria* bacterium SH2	*Acidobacteria*	KC715858.1	100
15	*Desulfomicrobium* sp.	*Proteobacteria*	JX548546.1	100
16	*Acidobacteria* bacterium D199A	*Acidobacteria*	KC845239.1	100
17	*Acidobacteria* bacterium S2-047	*Acidobacteria*	KF182983.1	99
18	*Mesotoga* sp. VNs100	*Thermotogae*	KC800693.1	100
19	*Pseudomonas* sp. clone S2P1061	*Proteobacteria*	KF145944.1	100
20	*Acidobacteria* bacterium SH6	*Acidobacteria*	KC715862.1	95
21	*Rhodospirillales* bacterium WX36	*Proteobacteria*	KC921187.1	98
22	*Acidovorax* sp. CPO 4.0017	*Proteobacteria*	KC902440.1	98
23	*Parvularcula* sp.REV_R1PII_12F	*Proteobacteria*	FJ933486.1	100
24	*Acidobacteria* bacterium CNY_02641	*Acidobacteria*	JQ402332.1	99
25	*Verrucomicrobia* bacterium	*Verrucomicrobia*	JF410432.1	99
26	*Nitrosomonas* nitrosa strain S12	*Proteobacteria*	KF483596.1	98

## Conclusions

A petrochemical wastewater treatment system combining an ICBAF unit, an HA unit, two A/O units, an MBR unit, and an O_3_-AC unit was proposed for treating influent with COD values exceeding 2500 mg L^−1^. Nineteen indexes were analyzed at seven sample points throughout the treatment process, with the results confirming the proposed process' ability to eliminate nearly all pollutants. In addition, the bacterial community in these units was verified, with *Proteosbacteria* and *Acidobacteria* being identified as the dominant bacteria.

## Methods

### Wastewater and characteristics

The PRWW treatment plant examined in this study was designed to treat wastewater generated by oil production processes in Guangdong Province, South China, at a rate of 300 m^3^ h^−1^. The wastewater processed by this plant is produced via processes such as crude electric desalting, crude oil tank drainage, and alkylation, and contains a significant number of pollutants, including refractory organic matter, ammonia nitrogen (NH_4_^+^-N), and heavy metals.

A schematic of the wastewater treatment plant and sampling locations is shown in Fig. 1. First, the wastewater undergoes physical and chemical pretreatment, which entails hydraulic retention times (HRT) of 3.5 h in the inclined plate sedimentation tank, the cavitation air flotation tank, and the dissolved air flotation tank. Next, the wastewater is fed through the internal circulation biological aerated filter (ICBAF) reactor for further biological strengthening pretreatment. In the ICBAF, the DO concentration is maintained at 3–4 mg L^−1^, the volume load is maintained at 3.94 kg (M^3^ D)^−1^, and the HRT is 14 h. After passing through the ICBAF, the wastewater flows into the hydrolysis acidification (HA) tank, where it stays for 25 h. Following the HA, the wastewater is then fed into the activated sludge system, which consists of two anaerobic and aerobic (A/O) biological treatment processes, for 24 h and 28.8 h, respectively. The sludge retention time in these two A/O units is 45 days. After A/O treatment, the wastewater undergoes membrane biological reactor (MBR) filtration, with an HRT of 3 h and and a DO concentration of between 3–4 mg L^−1^. Finally, the wastewater is fed into the ozone-activated carbon (O_3_-AC) unit before being discharged.

Wastewater samples were collected after each major step in this process, as indicated by S1 to S7 in Fig. 1.

### Analytical methods

The composition of organic pollutants in the influent and the concentrations of COD, BOD_5_, NH_4_^+^-N, nitrate nitrogen (NO_3_-N), nitrite nitrogen (NO_2_-N), total nitrogen (TN), total phosphorus (TP), and volatile fatty acid (VFA) were analyzed by using the Standard Methods^30^. The total organic carbon (TOC) content in the wastewater samples was measured using a TOC analyzer (astro TOC UV/Turbo, Hach Co. USA), while the concentrations of petroleum and animal and vegetable oil were determined via the infrared photo-metric method. The concentration of volatile phenol was analyzed using the 4-aminoantipyrine extraction spectrophotometry method, and the sulfide content was assessed via methylene blue spectrophotometry. Finally, the composition of organic matter in the wastewater was measured using a GC/MS analyzer (Thermo Finnigan SSQ710).

Methods for the semi-quantification of organics in wastewater have been reported previously^16^. Prior to use, the SPE column (Supelco Company, United States; extraction packing: octadecyl C18; weight 5 g; effective volume 20 mL) was pretreated by passing 10 mL of dichloromethane and 10 mL of ethyl acetate through it in order to remove impurities and allow the silica surface to be infiltrated more effectively. After pretreatment, the samples were loaded into the column, followed by dichloromethane to extract the organic compounds retained by the column. Finally, the extracts were evaporated and reconstituted to a volume of 1 mL using a 99.999% pure stream of nitrogen prior to analysis.

The stationary phase of the GC capillary column (60 m × 0.25 mm × 0.25 μm) was a HP-5MS neutral silica gel capillary. Analyses were carried out in a split ratio of 1:30, with an inlet temperature of 29 °C, and helium as a carrier gas with a flow rate of 1 mL min^−1^. The temperature was maintained at 60 °C for 10 min, before being increased to 270 °C at increments of 10 °C min^−1^, and then to 300 °C at increments of 20 °C min^−1^, where it was kept for 10 min. The MS detector was operated in electron ionization (70 eV) mode, with a scan range of 29–350 m z^−1^. Finally, the MS ion source temperature was set to 200 °C.

Dehydrogenase enzyme activity (DHA) was measured using the modified triphenyl tetrazolium chloride (TTC) dehydrogenase activity test^23^. This test utilized a 20 mL experimental mixture made up of the following components: 10 mL wastewater sample; 1 mL reactivated activated sludge(16 g/L); 4 mL 0.4% Tris–HCl (pH 8.4); 1 mL glucose (0.1 mol/L); 2 mL triphenyltetrazolium chloride (TTC) (0.4%); 1 mL Na_2_SO_3_ (0.36%) and 1 mL distilled water. The reactant was mixed thoroughly, and then left to sit for 120 min in an aqueous thermostat box at 37 ± 1℃ in the dark. After this period had elapsed, 1 mL of formaldehyde was added to the sample in order to stop the reaction. Finally, 5 mL acetone was added to the sample, which was followed by 10 min of shaking and 5 min of centrifugation at 4000 rpm. The absorbency of the supernatant was measured via a spectrophotometer at 485 nm and compared with a blank sample. The DHA was calculated according to the calibration curve for triphenyl formazan, with the average values being reported.

Polymerase chain reaction-denaturing gradient gel electrophoresis (PCR-DGGE) analysis was applied to analyze the microbial consortium in the ICBAF tank, the HA tank, the two A/O units, and the MBR tank, respectively. To this end, the procedure described by Xia et al^31^ was followed: (1) The DNA of the sludge samples was extracted using a Power Soil DNA Isolation Kit, and was examined using 1% agarose gel electrophoresis. (2) PCR amplification was performed using specifically synthesized primers, with the V3 and V4 regions of the 16S rDNA gene being selected for PCR. Specifically, the primers, 357F (5'-CCTACGGGAGGCAGCAG-3') and 518R (5'-ATTACCGCGGCTGCTGG-3'), were used. Replicate amplicons were pooled and visualized on 2.0% agarose gels using a Gel Doc XR system (Bio-Rad, US). (3) Amplicons of each sample were sent out for pyrosequencing by a commercial service (BGI, China), while the PCR products were sequenced by the Sangon Company (Shanghai, China). These sequences were then examined via BLAST (NCBI, USA) to identify and compare similarities.

## Data Availability

The datasets used and analysed during the current study are available from the corresponding author on reasonable request.
